# Prevalence of Dental Caries in Kosovar Adult Population

**DOI:** 10.1155/2016/4290291

**Published:** 2016-07-19

**Authors:** Blerim Kamberi, Ferit Koçani, Agim Begzati, Jeta Kelmendi, Donika Ilijazi, Nora Berisha, Lumnije Kqiku

**Affiliations:** ^1^Department of Dental Pathology and Endodontics, University Dentistry Clinical Center of Kosovo, 10000 Prishtina, Kosovo; ^2^Department of Pediatric and Preventive Dentistry, University Dentistry Clinical Center of Kosovo, 10000 Prishtina, Kosovo; ^3^Department of Orthodontics, University Dentistry Clinical Center of Kosovo, 10000 Prishtina, Kosovo; ^4^Private Dental Clinic, 10000 Prishtina, Kosovo; ^5^Division of Preventive and Operative Dentistry, Endodontics, Pedodontics and Minimally Invasive Dentistry, Department of Dentistry and Maxillofacial Surgery, University of Graz, 8010 Graz, Austria

## Abstract

*Objectives*. The aim of this study was to assess the prevalence of dental caries in the Kosovar adult population.* Materials and Methods*. A cross-sectional study in Kosovo was conducted examining 9387 patients, aged 18 upwards, between January 2010 and December 2011. Clinical evaluation was done using WHO criteria for evaluation of dental health status and data collection.* Results*. The prevalence of caries for the whole study was 72.80%. The mean DMFT index was 9.61 (±5.12) in the 18–34-year age group, 11.6 (±6.42) in the 35–44-year age group, 13.68 (±8.12) among the 45–64-year age group, 17.98 (±9.81) in the 65–74-year age group, and 23.19 (±9.41) in the age group of 75+ years, respectively. A significant difference of mean DMFT and its each component was observed between the ages (*P* < 0.001).* Conclusion*. This study comes out with the significant levels of dental caries among young Kosovar population (18–34 years old).

## 1. Introduction

Dental caries continues to be a major health concern for populations worldwide. Damage caused by caries leads to a decrease in the quality of life of the affected individuals and high economic costs for both individuals and society, with disparities related to well-known issues of socioeconomics, immigration, lack of preventive efforts, and dietary changes [1]. Despite the overall decline in caries prevalence in developed countries [2], caries continues to be an important disease in most developing countries. Studies have shown that caries remains a major problem in the adult population of both developing and industrialized countries [3–5].

Evaluation of the oral health status in the adult age group is important because it presents important information for planning services in dental care and also generates data on the outcomes of dental care provided to the population during their lifetime [6].

In Kosovo, no studies have been conducted which include examination by dentists to determine the prevalence of caries or tooth loss among adults. Most epidemiological studies have focused mainly on children rather than adults [7]. Therefore, strategies that are needed to treat affected people remain unknown. Data collected in this study may be useful to build a more complete profile of the oral health status of the adult population of Kosovo and will supplement the analysis of global trends of the disease.

Therefore, the objective of this study was to assess the prevalence and severity of dental caries in the Kosovar adult population in relation to their sex, age, and place of residence (urban or rural).

## 2. Materials and Methods

### 2.1. Subjects

This was a cross-sectional study comprising 9387 patients from different regions of Kosovo aged 18–75+. They were examined between January 2010 and December 2011 at the University Dentistry Clinical Center of Kosovo.

The University Dentistry Clinical Center of Kosovo is the only secondary and tertiary care center for the whole territory of Kosovo. Within each year, a large number of populations use the free services at the center, hence providing a heterogeneous population sample. It was due to the latter fact that the authors decided to use population samples from this center's dental records for this study.

### 2.2. Data Collection

Data were collected based on age, sex, and residence (rural or urban). The exclusion criteria were patients under the age of 18 and those who were missing any of the required information.

For calibration, six examiners were trained by an experienced dentist prior to the clinical examinations. The interexaminer agreement determined by Cohen's Kappa values was 0.7–0.9.

In the WHO criteria used for clinical evaluation of health status and data collection, only the evaluation of hard tissue of the teeth was included [8]. Patients were examined under an artificial light, using a dental mirror and a dental probe. All present teeth were taken into consideration during the clinical examination. Diagnosis of dental caries was made according to the criteria recommended by the WHO [8] (i.e., when a lesion in a pit or fissure or on a smooth tooth surface had an unmistakable cavity, undermined enamel, or a detectably softened floor or wall). Caries severity was measured by DMFT index, which records the number of DT (decayed teeth), MT (missing teeth), and FT (filled teeth).

### 2.3. Data Analysis

Clinical data were first entered into modified WHO Oral Health Status [8] paper forms, then entered into Excel, and subsequently exported to Statistical Package for Social Sciences (SPSS 17.0 for Windows, 13.0 Program Package) for statistical analysis. The prevalence proportion rates, mean values, and standard deviations were calculated for the purpose of analysis. Statistical analysis was performed applying descriptive statistics and the independent sample Chi-square test, Fisher Exact test between different groups, and one-way ANOVA tests to compare the means of DMFT, DT, MT, and FT to the other variables. The level of statistical significance was set at *P* = 0.05.

## 3. Results

**Figure 1:**
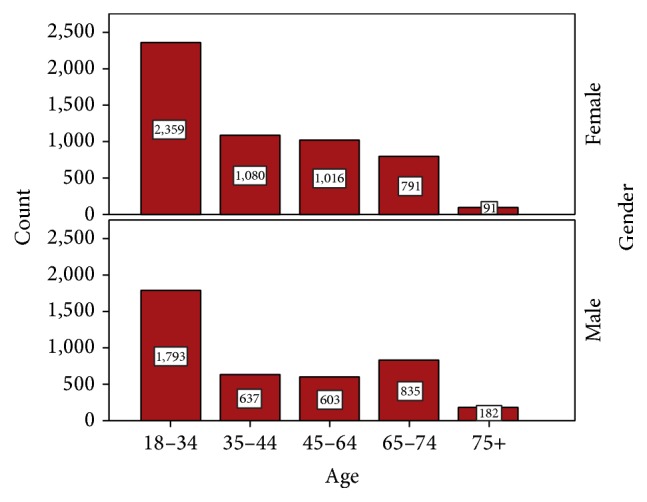
Patients participation according to sex and age group.

Figure 1 shows characteristics of the subjects by sex and group. The number of female participants was 5337 (59.5%), while the number of males was 4050 (40.5%).  Table 1 shows the subjects according to sex and residence. A total of 70.9% of subjects lived in the city while 29.1% lived in the countryside. According to sex, 73.3% of females and 67.6% of males lived in the city.

The highest percentage of participants diagnosed with caries was aged 18–34 years (4152 participants, 44.2%), while the lowest percentage was for participants aged 75 years and above (2.9%). The other age groups had similar participation rates: 18.3% for patients aged 35–44 years, 17.2% for patients aged 45–64 years, and 17.3% for patients aged 65–74 years.

Our data show significant differences between sex and residence and between sex and age (*P* < 0.001).  Table 2 shows the presence of caries according to sex, age, and residence.

According to the sex and caries distribution, 3903 patients (57.1%) with caries were female, while 2929 (42.9%) patients with caries were male.

Patients diagnosed with caries, 69.6%, lived in the city, compared with 30.4% who lived in the countryside. The age group with the highest prevalence of caries was the 18–34-year age group (54%), and the age group with the lowest prevalence was the age group of 75+ years (0.8%). Using the Chi-square test, we compared caries prevalence according to sex and found no significant difference for *P* = 0.354. However, when we compared caries prevalence according to age with caries prevalence according to residence, there was a significant difference in both comparisons for *P* < 0.001.

Table 3 shows the DMFT and caries prevalence according to age and sex. The prevalence of caries for the whole study was 72.80%, and it decreased with age: 18–34 years (88.80%), 34–44 years (77.90%), 45–64 years (65.50%), 65–74 years (42.40%), and 75+ years (20.90%). There was no significant difference between males and females.

The mean DMFT (SD) value for the study group was 12.52 (±7.91). The DMFT value increased with age from 9.61 (±5.12) for 18–34 years to 11.6 (±6.42) for 35–44 years, to 13.68 (±8.12) for 45–64 years, to 17.98 (±9.87) for 65–74 years, and to 23.19 (±9.41) for 75+ years.

The analysis of the DMFT components showed that the MT score was the major component.

These variables were also tested with ANOVA, and, as shown in the table for the DMFT index, there is no significant difference between the means according to sex (*P* = 0.613). There was also no significant difference between the means according to sex for the DT and MT components, but there was a significant difference between the mean values according to sex for the FT component (*P* < 0.001).

Our results show significant differences between the means according to age in the DMFT index and all of its components with *P* < 0.001.

Table 4 shows that caries prevalence was slightly higher for patients living in rural areas (75.9%) than for patients living in urban areas (71.5%).

The highest mean value and SD were for the MT component with a value of 7.31 ± 8.83, followed by the DT component with a mean value of 3.15 ± 3.31, and the FT component with the lowest mean value of 2.05 ± 2.86.

Comparing the mean values of the DMFT index and its components according to the patients' residence, we found no significant difference for the MT component with *P* = 0.617.

Patients from an urban residence had a higher DMFT index compared with those from a rural residence with a significant difference for *P* < 0.05.

Comparing the averages of the DT and FT components according to residence using one-way ANOVA test, we found that the average DT component was higher among the rural population (3.44 ± 3.45) than among the urban population (3.03 ± 3.24) with no significant difference for *P* < 0.001, although the average FT component was higher among the urban population (3.01 ± 2.26) than among the rural population (1.54 ± 2.41) with a highly significant difference for *P* < 0.001.

## 4. Discussion

Most studies about dental caries in Kosovo have focused on children under 12 years of age, and the state of dental caries in adults has remained unknown. A previous study assessing the prevalence and analyzing caries risk factors, in the Kosovar population between 2002 and 2005, had included measurements of early childhood caries, DEFT, and DMFT [7]. This is the first study that has investigated the epidemiology of dental caries among adults in Kosovo.

In agreement with most studies [9–11], it was found that the prevalence of caries was slightly higher in females than in males (Table 2). However, the present study did not observe any significant sex differences in caries prevalence that was confirmed in earlier findings [12, 13].

According to WHO recommendations, oral health surveillance of oral health conditions in adults should include the 35–44-year age group and the 65–74-year age group [8]. In our study, in addition to these two age groups, we included the age groups of 18–34 years, 45–64 years, and over 75 years (Figure 1). The prevalence of dental caries in the 18–34-year age group in the present study was found to be the highest. This outcome is thought to be a result of poor socioeconomic development, lack of preventive services in health insurance, and inadequate knowledge about the importance of oral health as a consequence of the absence of oral health promotion programs in adults.

The caries prevalence for the 35–44-year age group has been reported in many studies [14–16]. In the present study, the prevalence of dental caries in this age group was found to be 77.90%, which is lower than in some previous studies [14, 15, 17] but higher than in the studies conducted by Doifode et al. (48.6%) [16] and Namal et al. (58.2%) [5]. Our results showed that as age increased, the prevalence of caries decreased, which is in contrast with other research findings showing that as age increases so does the prevalence of caries [18]. This holds true since with age the prevalence of missing teeth increases compared to the prevalence of decayed and filled teeth.

Differences in the prevalence rates may be due to different study criteria. Observed differences in the caries prevalence of the adult population suggest that it may be possible to develop and implement oral health policies taking into account geographical and socioeconomic differences in populations [19].

Differences in the prevalence of caries between urban and rural areas are narrowing as the socioeconomic development in rural areas increases. Therefore, the prevalence of caries in some rural areas was even higher than in urban areas [20], which could be a result of lower level of oral health education and socioeconomic status. However, our study shows no significant difference in caries prevalence between urban and rural subjects (Table 4), which is in accordance with a study in the United States [21].

The mean DMFT for the study sample was 12.52 and increased among the ages (Table 3) and is consistent with the results of other studies [22, 23].

By the latest data, the mean DMFT value observed among 18–34-year age group was 9.61, compared to a value of 4.37 in an 18-25-year age group [24] in a neighbouring region, Italy, and 12.76 in a 20-34-year age group [19] in Hungary; our score shows moderate condition among this population, which means a relative “average position” for Kosovo.

In the 35–44-year age group, the mean DMFT value was 11.6, which was a better score than in many countries; DMFT scores for the same age group were 16.1 in Germany [25], 15.4 in Belgium [26], 14.7 in Austria [27], 15.4 in Hungary [19], and 14.4 in Norway [28]. However, the mean DMFT score in our study for individuals aged 65–74 years (17.98) was higher than in many other countries [19, 25, 27].

Comparisons of the mean DMFT findings with those of other studies should be undertaken with caution, since there is great variation in the findings in the literature. This discrepancy may be due to differences in geographic location, population culture, diagnostic criteria, and sampling procedures.

The value of F/DMFT observed in our study was 2.47 for the 35–44-year age group and only 0.77 for the 65–74-year age group. These values are significantly lower than those in certain European countries such as Spain, Denmark, Austria, and France, where patients have a higher proportion of fillings than in Kosovo [17, 27, 29, 30]. A potential reason for this might be that people in Kosovo tend to seek dental care when they experience severe dental pain rather than for routine dental checkups; hence, by the time they seek dental care, the caries is at an advanced stage. In most cases, the tooth has to be extracted because it is beyond restoration or requires complex and expensive endodontic treatment. The high percentage of untreated teeth in individuals aged 65–74 years may be due to difficulties in accessing dental services and a lack of interest in dental health or due to other health conditions.

It was also noted that the DT component decreased with age while the MT component increased with age. Reasons for the high prevalence of decayed teeth in adults aged 18–34 years, together with the low prevalence of filled teeth in comparison with other European countries, are mainly a consequence of the health insurance system in Kosovo.

Fillings and endodontic treatment are free for about 40% of the Kosovar population covered by the private health insurance system, which still does not include free preventive measures and guidelines for adults. In summary, the high prevalence of decayed, missing, and filled teeth in the adult population of Kosovo also reflects the lack of preventive services in the state health insurance system.

There is a high DT component and there is a very low FT component of the DMFT, mainly because of the lack of restorative dental services or to some extent low utilization of this service.

As a consequence of the absence of the data, we were not able to compare our results with neighboring states.

## 5. Conclusion

The present study demonstrated significant levels of dental caries among young Kosovar population (18–34 years old) where carious and missing teeth due to caries are predominant.

It is understandable that Kosovo, as a new state with major economic and social problems and with a developing health care department, has a significant number of priorities to balance with the dental-oral health care of the population. Therefore, based on the WHO recommendations, to improve the general oral health of the population, the focus should be on prevention, starting with health education, promotion of oral health, and application of preventive measures in the Kosovar population.

Further studies of dental health that cover more regions of Kosovo will help to identify public dental health problems, an essential step in improving the general health status of the citizens of this country [31].

## Figures and Tables

**Table 1:** Patients participation according to sex and residence.

Sex	F		M		Total		*P* value
	*N*	%	*N*	%	*N*	%	
Residence							
Rural	1425	26.7	1311	32.4	2736	29.1	*P* < 0.001
Urban	3912	73.3	2739	67.6	6651	70.9	

**Table 2:** Caries presence according to gender, age, and residence.

Caries	No		Yes		Total		*P* value
	*N*	%	*N*	%	*N*	%	
Gender							
Female	1434	56.1	3903	57.1	5337	56.9	*P* = 0.354
Male	1123	43.9	2927	42.9	4050	43.1	

Age group							
18–34	467	18.3	3685	54	4152	44.2	*P* < 0.001
35–44	380	14.9	1337	19.6	1717	18.3	
45–64	558	21.8	1061	15.5	1619	17.2	
65–74	936	36.6	690	10.1	1626	17.3	
75+	216	8.4	57	0.8	273	2.9	

Residence							
Rural	659	25.8	2077	30.4	2736	29.1	*P* < 0.001
Urban	1898	74.2	4753	69.6	6651	70.9	

**Table 3:** DMFT, D, M, and F and caries prevalence according to sex and age group.

	DMFT		D		M		F		Caries prevalence
	Mean	SD	Mean	SD	Mean	SD	Mean	SD	
Sex									
Female	12.56	7.682	3.25	3.339	7.1	8.538	2.21	2.962	73.10%
Male	12.47	8.206	3.03	3.268	7.6	9.214	1.84	2.725	72.30%

Total	12.52	7.912	3.15	3.31	7.31	8.839	2.05	2.868	72.80%

		*P* = 0.613		*P* = 0.002		*P* = 0.007		*P* < 0.001	

Age group									
18–34	9.61	5.123	4.48	3.528	2.47	3.161	2.66	3.129	88.80%
35–44	11.6	6.425	3.18	3.087	5.95	5.878	2.47	3.067	77.90%
45–64	13.68	8.125	2.2	2.607	9.85	8.546	1.63	2.479	65.50%
65–74	17.98	9.876	1.14	1.923	16.06	10.8	0.77	1.688	42.40%
75+	23.19	9.419	0.53	1.453	22.35	10.095	0.32	0.972	20.90%

Total	12.52	7.912	3.15	3.31	7.31	8.839	2.05	2.868	72.80%

		*P* < 0.001		*P* < 0.001		*P* < 0.001		*P* < 0.001	

**Table 4:** DMFT, D, M, and F and caries prevalence according to residence.

	DMFT		D		M		F		Caries prevalence
	Mean	SD	Mean	SD	Mean	SD	Mean	SD	
Residence									
Rural	12.23	7.862	3.44	3.459	7.24	8.631	1.54	2.411	75.90%
Urban	12.64	7.93	3.03	3.24	7.34	8.924	2.26	3.011	71.50%

Total	12.52	7.912	3.15	3.31	7.31	8.839	2.05	2.868	72.80%

		*P* = 0.022		*P* < 0.001		*P* = 0.617		*P* < 0.001	
